# Phase I study evaluating the safety, tolerability and pharmacokinetics of a novel oral dissolvable film containing dexamethasone versus Fortecortin dexamethasone tablets

**DOI:** 10.1080/20018525.2017.1353395

**Published:** 2017-08-03

**Authors:** Zuzana Diamant, Gabriella Samuelsson Palmgren, Bengt Westrin, Leif Bjermer

**Affiliations:** ^a^ Department of Respiratory Medicine & Allergology, Institute for Clinical Science, Skåne University Hospital, Lund, Sweden; ^b^ Department of Clinical Pharmacy & Pharmacology, General Practice & QPS-NL, Groningen, The Netherlands; ^c^ Clinical Trial Unit, Clinical Studies Sweden- Forum South, Skåne University Hospital, Lund, Sweden; ^d^ AcuCort AB, Helsingborg, Sweden

**Keywords:** Dexamethasone, drug formulation, phase 1 clinical trials, pharmacokinetics, allergy, anaphylaxis

## Abstract

**Introduction**: Systemic corticosteroids are anti-inflammatory agents with dexamethasone among the most potent in the class. Within (respiratory) allergy, systemic corticosteroids are usually applied in medical emergencies. In these situations, patients may experience physical or logistic problems taking tablets. To fulfil a practical unmet need for outpatients, Dexa ODF, an oral dissolvable film containing dexamethasone, was developed.

**Objectives**: We compared the safety, tolerability and pharmacokinetics (PK) of Dexa ODF with Fortecortin tablets in healthy subjects.

**Methods**: Thirty subjects participated in this open label, two-way, cross-over study, consisting of two treatment visits separated by 5–10 days. On both treatment visits, subjects randomly received one single dose of Dexa ODF (one strip; 8 mg dexamethasone) or one single dose of Fortecortin (two 4 mg tablets). Safety evaluations and blood sampling for PK were conducted until 48 h post-dose and bioequivalence analysis was performed on AUC(0-t), AUC(0-∞) and Cmax.

**Results**: All subjects were dosed. Forty-five adverse events (AEs) were reported by 17 subjects and approximately 50% were deemed ‘possibly treatment related’ (14 on Dexa ODF; 12 on Fortecortin) with no significant difference between treatments. For all three bioequivalence parameters the 90% CIs were within the acceptance limits of bioequivalence (0.8;1.25).

**Conclusion**: We demonstrated good tolerability and bioequivalence of Dexa ODF (8 mg dexamethasone) compared to Fortecortin tablets (2 × 4 mg dexamethasone). Dexa ODF is currently under development as an innovative treatment for use within respiratory and allergic conditions, including emergencies.

## Introduction

Systemic corticosteroids are potent anti-inflammatory agents that are being used in several therapeutic areas for over 70 years. Within this class, several drugs with different potencies and durations of action have been developed. Among these, hydrocortisone is short-acting and the least potent, prednisone, prednisolone and methylprednisolone are intermediate-acting and intermediate-potent, while dexamethasone provides long-lasting effectiveness with a potency approximately four to five times higher than ‘the intermediate group’.[1,2]

Therapeutic indications for systemic corticosteroids vary from acute (rescue) use, as in allergic and respiratory emergencies, to maintenance or adjuvant therapy as, for instance, in chronic inflammatory and parenchymal lung diseases, Addison’s disease, brain edema, autoimmune diseases, transplantation medicine and as treatment for chemotherapy-induced nausea and vomiting (CINV).[3–8]

According to guidelines, oral corticosteroids are advocated to treat severe asthma exacerbations, especially in general practice.[9] During exacerbations or other allergic emergencies, patients may experience problems with taking currently available oral medications, for example because no water is available or because of severe distress. In non-acute situations, and particularly for CINV patients, swallowing tablets may also impose a problem because the patient is nauseous or in a terminal stage.

As a solution to these medical conditions, and to fulfill a practical unmet need, a patient-friendly formulation consisting of an oral dissolvable film (ODF) containing dexamethasone has been developed. The choice of the corticosteroid for this product was based on its potency, allowing applications both in acute allergic conditions (including anaphylaxis) and respiratory emergencies as well as in more chronic conditions such as CINV, brain metastasis and edema. These applications typically require single doses of dexamethasone of 4–8 mg or higher and hence, 8 mg was chosen for this product but it can also be produced in other strengths, e.g. 4 mg.

Here, we report a phase I study comparing the pharmacokinetic profiles, safety and tolerability of a single dose of Dexa ODF (one strip of 8 mg dexamethasone) with a single dose of the registered dexamethasone tablet Fortecortin (two tablets of 4 mg dexamethasone each) in healthy subjects.

## Subjects and methods

### Subjects

Thirty healthy male subjects participated in this study. All subjects declared being in good general health without any history of clinically relevant disorders, with no clinically relevant abnormalities on physical examination, vital signs, routine laboratory and ECG at screening. Main exclusion criteria consisted of any relevant prior medical condition, prior history of abuse of drugs, tobacco or alcohol, relevant (drug) allergies, use of any concomitant medications (including herbals, vitamins or minerals) and recent intake of cytochrome P450 3A4-interacting agents (e.g. grapefruit or orange containing foods).

This single center study was conducted at the Clinical Trial Unit of Skane University Hospital in Lund, Sweden, and had been approved by the Regional Ethical Review Board as well as by the Swedish Medical Products Agency. All subjects provided written informed consent before enrolment into the study. All study-related procedures were conducted in accordance with the Declaration of Helsinki and the International Conference of Harmonization (ICH) Guideline for Good Clinical Practice. The study was registered under EUDRACT number 2013–001730-18.

### Study design

**Figure 1.**
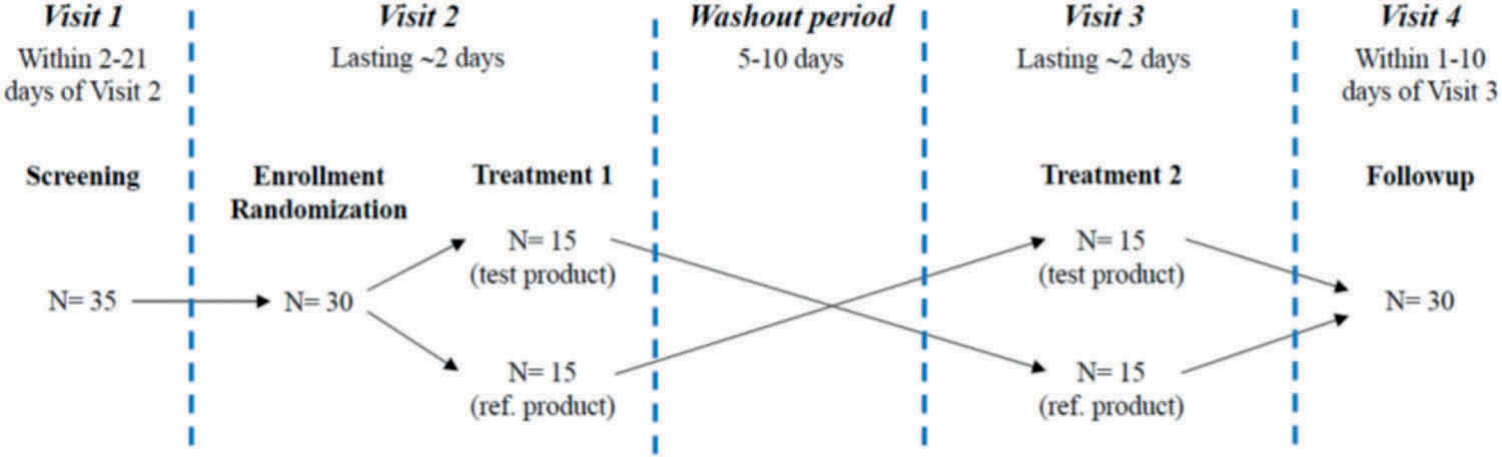
Study design.

This study had a randomized, open label, two-way, cross-over design and consisted of a screening visit (visit 1) to test subjects’ eligibility, followed by two treatment visits (visit 2 and visit 3, respectively) separated by a washout period of 5–10 days. There was a follow-up visit (visit 4) after study finalization (Figure 1). On both treatment visits, subjects randomly received a single dose of the study medication (i.e. Dexa ODF or Fortecortin) under fasting conditions. Blood samples were drawn at predefined time points from pre-dose until 48 h post-dose; in parallel, adverse events were recorded and safety assessments were performed.

### Study medication and dose selection

#### Test product

The test product, Dexa ODF (batch 7,018,863), is being developed by AcuCort AB, Helsingborg, Sweden. Dexa ODF is a thin oral dissolvable film, sized 20 × 33 × 0.025 mm, containing 8 mg dexamethasone. Apart from micronized dexamethasone, Dexa ODF contains the inactive ingredient hydroxypropyl methylcellulose (HPMC). When placed on the tongue, Dexa ODF rapidly dissolves in saliva and the active ingredient becomes available for systemic absorption.

#### Reference product

Fortecortin is a dexamethasone tablet that has been approved for marketing in the EU. It is available in strengths of 1, 4 and 8 mg (Merck, S.L., Madrid, Spain). In this study, two Fortecortin tablets (batch M1337) of 4 mg each were administered as the reference product and swallowed with a standardized volume of 150 ml water.

In the test product (Dexa ODF, batch 7018863), the content of dexamethasone was determined at 8.152 mg per strip. For the reference product (Fortecortin, batch M1337), the content of dexamethasone was determined at 4.035 mg per tablet. Thus, two tablets of Fortecortin administered to participants in this study yielded a total dose of dexamethasone of 8.070 mg with an overall difference in dexamethasone content between test and reference products of <1%.

According to Fortecortin’s SmPC (Summary of Product Characteristics), the recommended initial doses for adults with acute severe asthma are 8–20 mg and up to 48 mg.

### Methods

During the treatment visits (visit 2 and 3), subjects were fasted overnight for at least 8 h pre-dose until at least 4 h post-dose. Standardized meals were provided at pre-set timepoints, 4 and 10 h post-dose. During each treatment visit, blood samples for PK analysis were drawn from a cannula placed in the fore-arm at pre-dose (i.e. within 1 h pre-dose) and at 15, 30, 45, 60, 75, 90 and 105 min and at 2, 2.5, 3, 5, 8 and 12 h (before discharge from the unit) post-dose, and subsequently, at single ‘come back’ occasions at 24, 36 and 48 h (±1 h) post-dose. In parallel, safety assessments were performed repeatedly by adverse events recordings, physical examination, vital signs measurements, safety laboratory tests and ECGs. Adverse events were reported using MedDRA terminology.

### Blood samples processing

Blood samples were centrifuged at approximately 3500 rpm for 10 min at 2–8°C. The plasma was carefully removed using a pipette and stored in aliquots at −20°C, within 4 h transferred to −80°C, pending analysis. The concentration of dexamethasone in the plasma samples was determined using a validated ultra-performance liquid chromatographic-tandem mass spectrometric (UPLC-MS/MS) method. The bioanalytical method validation included selectivity, linearity, precision, accuracy, dilution integrity, matrix effects and stability issues. The development, validation and execution of this bioanalytical method were conducted at a GLP certified contract laboratory (the National Veterinary Institute, SVA, Uppsala, Sweden).

### Statistical analysis

All 30 subjects were included in the statistical evaluation. No adjustment for multiple testing was done. The sample size of 30 subjects was based on previous findings from current literature.[9] Based on intra-subject coefficients in this study, a sample size of 8 and 22 subjects was required to show bioequivalence at 90% power and acceptance limits of 80–125% for AUC and Cmax, respectively. Safety and tolerability were reported using MedDRA terminology and summarized by treatment group using descriptive statistics.

Pharmacokinetic analysis was performed by Scandinavian Development Services AB, Danderyd, Sweden. Pharmacokinetic parameters (tmax, Cmax, T1/2, AUC(0-t) and AUC(0-∞)) for dexamethasone were calculated from plasma samples obtained from subjects during both dosing periods using standard non-compartmental methods. Descriptive statistical analysis for pharmacokinetic variables was based on (i) subject listings, (ii) graphs and (iii) summary statistics comprising geometric mean, coefficient of variation, arithmetic mean, standard deviation, median, minimum and maximum, as appropriate. The coefficient of variation (CV,%) was calculated as:

(1)
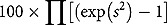

with ‘s’ as the standard deviation of the data on a log scale. The computational software used for the statistical analysis was SAS®, version 9.3 (SAS institute inc. NC, USA).

Differences in PK parameters of both dexamethasone formulations (i.e. log-transformed ratios of AUC(0-t), AUC(0-∞) and Cmax, and tmax) were tested using an analysis of variance (ANOVA) or a non-parametric test, as appropriate. The ANOVA model included ‘sequence’, ‘subject within sequence’, ‘period’ and ‘formulation’ as fixed effects. An F-test was used to investigate whether or not Cmax for Dexa ODF was lower than for Fortecortin, using log-transformed ratios of Cmax. The test was two-sided at significance level 5% (alpha = 0.05). Wilcoxon paired signed rank test was used to compare tmax for Dexa ODF versus Fortecortin. The test was one-sided at significance level 5% (alpha = 0.05).

Additionally, AUC (0-t), AUC (0-∞) and Cmax were tested for bioequivalence between formulations. If the 90% confidence intervals for the log-transformed ratios of AUC(0-t) were within acceptance limits 80–125%,[10] based on the back-transformed lower and upper limits, this was interpreted as bioequivalence between the two products.

## Results

### Safety and tolerability

Thirty-five healthy subjects were screened and 30 of them were randomized. All randomized subjects completed the study and were dosed both Dexa ODF and Fortecortin (Table 1). In all, 17 subjects reported 45 AEs (26 AEs within Dexa ODF administration, including two at follow-up; 19 AEs within Fortecortin administration). Most frequent AEs consisted of headache, fatigue and polyuria. Approximately 50% of AEs were deemed ‘possibly treatment related’ (14 on Dexa ODF and 12 on Fortecortin) with no significant difference between treatments. Forty-four AEs were self-limiting and mild in intensity, while one AE was reported moderate (neck pain, treatment unrelated). There were no clinically relevant changes or trends observed in vital signs, laboratory variables or ECGs in any of the subjects during the study.

**Table 1.** Demographics of randomized subjects.

Demographics	*n* = 30
**Age (years)**	
*Mean*	25.2
*SD*	4.0
*Median*	25
*Ranges*	19–37
**Race (*n*; %)**	
*Caucasian*	29 (96.7%)
*Asian*	1 (3.3%)
**Weight (kg)**	
*Mean*	73.4
*SD*	8.1
*Median*	72.8
*Ranges*	56.2–87.7
**Height (m)**	
*Mean*	1.80
*SD*	0.07
*Median*	1.80
*Ranges*	1.70–1.96
**BMI (kg m^–^^2^)**	
*Mean*	22.6
*SD*	2.1
*Median*	22.5
*Ranges*	18.0–26.8

### Pharmacokinetics and bioequivalence testing

**Figure 2.**
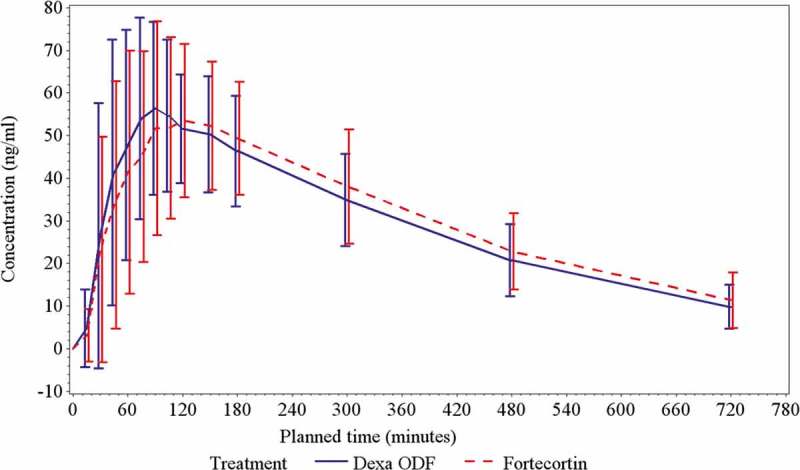
Mean (± SD) dexamethasone plasma concentration over time during both treatments.

Figure 2 shows the mean plasma concentrations over time for both the test and reference product and Table 2 shows a summary of the PK parameters for both study formulations. No statistical difference was found between Dexa ODF and Fortecortin in any PK parameter, but Dexa ODF appeared to have a faster absorption rate compared to Fortecortin (mean tmax 87 vs. 107.6 min, respectively), but failed to reach statistical significance (p = 0.0575). In addition, PK parameters AUC(0-t), AUC(0-∞) and Cmax met the acceptance limits of bioequivalence (i.e. all 90% CIs within 80–125%) (Table 3).

λ_z_ = terminal rate constant

**Table 2.** Pharmacokinetic parameters following single doses of Dexa ODF and Fortecortin in healthy subjects.

Treatment	Parameter (unit)	n	Mean	SD	Median	Min	Max	Geometric mean	CV (%)
Dexa ODF 8 mg	AUC_0-t_ (min*ng ml^–1^)	30	24,490.0	8236.2	23,257.0	11,983.9	42,786.2	23,235.5	33.8
	AUC_0-∞_ (min*ng ml^–1^)	30	24,764.7	8263.8	23,650.4	12,154.0	43,266.8	23,514.2	33.5
	C_max_ (ng ml^–1^)	30	72.7	19.1	69.1	38.7	118.2	70.3	26.8
	t_max_ (min)	30	87.0	41.7	75.0	30.0	180.0	78.0	51.0
	t½ (min)	30	251.9	52.6	236.9	141.7	416.1	246.9	20.6
	λ_z_	30	0.00286	0.00059	0.00293	0.00167	0.00489	0.00281	20.6
Fortecortin 2 × 4 mg	AUC_0-t_ (min*ng ml^–1^)	30	26,032.5	9294.8	23,702.1	11,461.2	53,570.5	24,591.1	35.1
	AUC_0-∞_ (min*ng ml^–1^)	30	26,345.0	9322.1	24,174.1	11,597.4	54,024.4	24,906.4	34.9
	C_max_ (ng ml^–1^)	30	73.0	18.3	75.0	33.8	110.3	70.5	28.7
	t_max_ (min)	30	107.6	55.1	98.0	30.0	300.0	95.2	55.0
	t½ (min)	30	253.9	44.7	252.9	190.4	400.8	250.5	16.4
	λ_z_	30	0.00280	0.00043	0.00274	0.00173	0.00364	0.00277	16.4

**Table 3.** Comparison of PK parameters of Dexa ODF 8 mg versus Fortecortin 2 × 4 mg in healthy subjects.

Treatment comparison Dexa ODF 8 mg versus Fortecortin 2 × 4 mg	Parameter (unit)	n	Geometric mean (ratio)	90% Confidence Interval	p-value
	AUC_0-t_ (min*ng ml^–1^)	30	0.945	(0.897, 0.995)	
	AUC_0-∞_ (min*ng ml^–1^)	30	0.944	(0.898, 0.993)	
	C_max_ (ng ml^–1^)	30	0.998	(0.914, 1.089)	two-sided: 0.964
	t_max_ (min)	30		Dexa<Forte	one-sided: 0.0575

## Discussion

In this study, we evaluated and compared the safety, tolerability and pharmacokinetics (PK) of Dexa ODF, an innovative dexamethasone formulation, with Fortecortin, a dexamethasone tablet approved for marketing in the EU, in 30 healthy males. Both products were generally well-tolerated, and no safety issues occurred during the study. Although slightly more AEs were reported after Dexa ODF administration, these were all mild and self-limiting with only a minority deemed to be ‘possibly treatment related’, with no significant difference between the two products. Both products showed a similar PK profile and bioequivalence was met according to conventional criteria for AUC and Cmax.

Our data complement the findings from a previous study by Nishigaki et al. comparing the clinical efficacy of another dexamethasone oral dissolvable film with a marketed dexamethasone tablet in nauseous patients with breast cancer following chemotherapy.[11] In that study, both dexamethasone products were administered at 8 mg daily dose 2–4 days after combination chemotherapy. Although both products offered a similar anti-emetic efficacy, the patients preferred the oral dissolvable film over the tablets due to its user friendliness and better taste.[11]

To further improve patient-friendliness for both children and adults across different clinical indications, Dexa ODF has also been developed in a 4 mg strength corresponding to approximately 25 mg of prednisolone. The Dexa ODF strength of 8 mg used in the current study thus corresponds to approximately 50 mg of prednisolone.

Additionally, we compared the AUC(0-t) and Cmax from our study with previously published PK data. Following a PubMed search, we found eight human studies reporting AUC(0-t) and Cmax for orally administered dexamethasone doses between 0.5 mg and 300 mg.[12–19] Although two of these studies [12,18] reported deviating results for various reasons, overall, it was concluded that the pharmacokinetics of oral dexamethasone show linearity and that the AUC and Cmax obtained for Fortecortin (and Dexa ODF) in the current study fit in with current literature.

## Conclusion

In summary, we report a good safety and pharmacokinetics of an innovative dexamethasone oral product currently under development for both children and adults across several clinical indications including anaphylaxis, allergic syndromes, acute (asthmatic, COPD or croup-related) airway obstruction and as supportive therapy in debilitating conditions, such as cancer. Due to its favorable pharmacokinetic profile, it can be a more patient-friendly alternative to conventional oral tablets of dexamethasone, prednisolone and other glucocorticoids in particular for outpatients with allergic or respiratory emergencies or in nauseous patients.
